# Association between the geriatric nutritional risk index and postoperative delirium: a meta-analysis

**DOI:** 10.3389/fpsyt.2026.1826824

**Published:** 2026-04-23

**Authors:** Dandan Han, Yingsi Liang, Qian Chen, Xinyu Wang, Xiao Chen

**Affiliations:** 1 Anesthesiology Department, Women’s Hospital, School of Medicine Zhejiang University, Hangzhou, China; 2 Jiangsu Province Key Laboratory of Anesthesiology, Xuzhou Medical University, Xuzhou, China; 3 Wuxi Medical College, Jiangnan University, Wuxi, China

**Keywords:** geriatric nutritional risk index, malnutrition, meta-analysis, postoperative delirium, risk factor

## Abstract

**Background:**

Malnutrition has been suggested as an important contributor to postoperative delirium (POD). The geriatric nutritional risk index (GNRI), a simple indicator based on serum albumin and body weight, has been increasingly evaluated in surgical patients. This meta-analysis aimed to clarify the association between GNRI and the risk of POD.

**Methods:**

PubMed, Embase, and Web of Science were searched for observational studies evaluating the association between categorized GNRI and POD. Pooled risk ratios (RRs) and 95% confidence intervals (CIs) were calculated using random-effects models accounting for heterogeneity.

**Results:**

Twelve cohort studies involving 12,332 surgical patients were included. Overall, low GNRI was associated with a significantly increased risk of POD (RR = 1.62, 95% CI: 1.34–1.96; I² = 27%). Subgroup analyses showed a stronger association in studies with mean patient age > 74 years compared to ≤ 74 years (*p* for subgroup difference = 0.02) and in those using GNRI cutoffs < 98 compared to ≥ 98 (*p* = 0.04). The association was weaker in studies using multivariate analyses compared with univariate analyses (*p* = 0.01). Meta-regression indicated that GNRI cutoff values significantly influenced the pooled effect (*p* = 0.04). The funnel plot showed mild asymmetry, whereas Egger’s test was not significant (*p* = 0.11), and trim-and-fill analysis produced a similar pooled estimate (RR = 1.54, 95% CI: 1.26–1.87).

**Conclusions:**

Low GNRI is associated with an increased risk of POD in surgical patients, suggesting that preoperative nutritional assessment may help identify individuals at higher risk for this complication.

**Systematic Review Registration:**

The study protocol was registered prospectively in the PROSPERO database (registration number: CRD420261335609).

## Introduction

Postoperative delirium (POD) is an acute neuropsychiatric syndrome characterized by fluctuating disturbances in attention, awareness, and cognition that develop within a short period after surgery (1, 2). It is typically diagnosed using standardized criteria or assessment tools such as the Diagnostic and Statistical Manual of Mental Disorders (DSM) or the Confusion Assessment Method (CAM) (3). POD is one of the most common postoperative complications, particularly among older surgical patients, with reported incidence ranging from approximately 10% to over 50% depending on patient characteristics and the type of surgery (4, 5). The occurrence of POD is clinically important because it is associated with multiple adverse outcomes, including prolonged hospitalization, increased healthcare costs, higher risks of postoperative complications, long-term cognitive decline, institutionalization, and increased mortality (6–8). Although several traditional risk factors for POD have been identified—such as advanced age, preexisting cognitive impairment, comorbidities, and surgical stress—these factors alone do not fully explain the variability in POD occurrence (9). Therefore, identifying additional and potentially modifiable risk factors has become an important focus of current perioperative research to improve risk stratification and guide preventive strategies.

Malnutrition has increasingly been recognized as a key contributor to adverse surgical outcomes and may also play an important role in the development of POD (10). Several biological mechanisms may plausibly link poor nutritional status to delirium. Malnutrition is associated with systemic inflammation, immune dysregulation, oxidative stress, and impaired metabolic reserve, all of which may increase vulnerability of the brain to perioperative insults (11, 12). In addition, inadequate protein and micronutrient intake can lead to hypoalbuminemia, altered neurotransmitter synthesis, and reduced antioxidant capacity, potentially contributing to neuroinflammation and disruption of neuronal function (12). The geriatric nutritional risk index (GNRI) is a simple and objective tool developed to evaluate nutritional risk in hospitalized patients, particularly older adults (13). It is calculated based on serum albumin concentration and the ratio of actual body weight to ideal body weight, providing an integrated measure of protein status and body composition (13). Because these variables are routinely measured in clinical practice, GNRI offers a convenient approach for perioperative nutritional assessment (14). In recent years, several observational studies have investigated the association between GNRI and the risk of POD in surgical populations (15–26). However, the results of these studies have been inconsistent, possibly due to differences in study design, patient characteristics, GNRI cutoff values, and analytical approaches (15–26). Therefore, a comprehensive synthesis of the available evidence is needed. The present meta-analysis aimed to systematically evaluate the association between GNRI and the incidence of POD in surgical patients and to explore potential sources of heterogeneity among the included studies.

## Methods

The meta-analysis was carried out in accordance with established methodological guidance, following the principles outlined in the PRISMA 2020 statement (27) and the Cochrane Handbook for Systematic Reviews and Meta-Analyses (28), encompassing protocol planning, study selection, data extraction, statistical analysis, and reporting. The study protocol was registered prospectively in the PROSPERO database (registration number: CRD420261335609).

### Database search

We carried out a comprehensive literature search across PubMed, Embase, and Web of Science to identify eligible studies for inclusion. The search strategy was constructed using three core concept domains (1): “geriatric nutritional risk index” OR “geriatric nutrition risk index” OR “GNRI”; (2) “surgery” OR “surgical” OR “postoperative” OR “postsurgical”; and (3) “confusion” OR “delirium” OR “acute encephalopathy” OR “cognitive dysfunction” OR “cognitive impairment” OR “cognitive disorder” OR “altered mental status” OR “organic brain syndrome” OR “acute encephalopathy”. Only full-text, peer-reviewed articles published in English and conducted in human populations were considered eligible. We also manually examined the reference lists of relevant reviews and original studies to capture additional potentially eligible reports. Each database was searched from inception through 26 January 2026. The complete search strategies for all databases are provided in **Supplementary File 1**.

### Study inclusion and exclusion criteria

The selection of studies was guided by the PICOS principle:

Population (P): Adult patients (≥ 18 years) undergoing any type of surgical procedure under general or regional anesthesia. Studies enrolling mixed populations were included if data for adult surgical patients could be clearly identified.

Exposure (I): GNRI assessed during the perioperative period and analyzed as a categorized variable, with the lowest GNRI category (indicating poorer nutritional status) compared with the highest GNRI category.

Comparator (C): Patients with higher GNRI categories (better nutritional status) served as the reference group when estimating the association between GNRI and POD.

Outcome (O): The primary outcome was the incidence of POD diagnosed during the postoperative period using validated diagnostic criteria or assessment tools, such as the CAM, the DSM, International Classification of Diseases codes, clinical diagnosis as evidenced by medical records, or other clinically accepted definitions consistent with those reported in the original studies.

Study design (S): Observational studies with longitudinal follow-up, such as prospective or retrospective cohort studies, nested case–control studies, and *post-hoc* analyses of clinical trials.

The studies were excluded according to the following criteria: (1) reviews, meta-analyses, editorials, letters, case reports, or case series; (2) studies that did not evaluate the association between GNRI and POD; (3) studies in which GNRI was analyzed only as a continuous variable without categorized comparisons; (4) studies that did not involve adult patients undergoing surgery; (5) studies in which POD was not clearly defined or reported; (6) studies that did not provide extractable effect estimates or sufficient data for calculation; (7) duplicate or overlapping study populations, in which case the study with the largest sample size or the most complete data was included; and (8) animal or experimental studies.

### Study quality evaluation and data extraction

Two reviewers independently performed the literature search, screened eligible studies, assessed study quality, and extracted relevant data. Any disagreements were resolved through discussion, and when necessary, by consulting the corresponding author. Study quality was appraised using the Newcastle–Ottawa Scale (NOS) (29). The NOS examines methodological rigor across selection, comparability, and outcome ascertainment domains. Total scores vary from 1 to 9, and studies achieving ≥ 8 points were considered to be of high quality. Extracted data included study characteristics (first author, publication year, country, and study design), participant characteristics (sample size, age, sex, and type of surgery), exposure assessment (timing for measuring GNRI, and cutoff of GNRI for defining malnutrition risk), follow-up duration, outcome validation (methods for the diagnosis of POD, and numbers of patients who developed POD), and covariates included in the adjusted analyses examining the association between GNRI and POD.

### Statistical analyses

The association between malnutrition as indicated by low GNRI and the incidence of POD in adults was evaluated by combining RRs and their corresponding 95% CIs, comparing individuals with the lowest vs. the highest category of GNRI in each study. When necessary, effect estimates and standard errors were derived from reported 95% CIs or *p* values. All estimates were log-transformed before pooling to enhance normal distribution assumptions and stabilize variances (28). Hazard ratios (HRs) were considered approximately equivalent to RRs in cohort studies with relatively low event rates (28). For studies reporting odds ratios (ORs), these were converted to RRs using an established method based on the baseline risk (P_0_) in the reference group, according to the formula: RR = OR/([1 − P_0_] + [P_0_ × OR]) (30). To evaluate variability across studies, we applied the Cochrane Q test and calculated the I² statistic (31). I² values below 25% were classified as low heterogeneity, 25–75% as moderate, and above 75% as high heterogeneity. Pooled effect estimates were calculated using a random-effects model to accommodate variability across studies (28). We performed leave-one-out sensitivity analyses, sequentially excluding individual studies to assess the robustness of the findings (32). To identify possible sources of between-study variability, we performed predefined subgroup analyses stratified by study design (prospective or retrospective), mean ages, sex distribution, cutoffs of GNRI, average follow-up length, methods for the diagnosis of POD (clinical assessment vs. medical records evidenced diagnosis), analytic models (univariate vs. multivariate), and NOS score. Continuous variables were dichotomized using their median values as cutoffs to balance the number of studies in each subgroup. These thresholds were not based on predefined clinical criteria and were applied for exploratory purposes. In addition, the univariate meta-regression analysis was performed to evaluate the influence of study characteristics in continuous variables on the outcome, such as mean ages, proportions of men, cutoffs of GNRI, follow-up durations, and NOS scores. To assess potential publication bias, we inspected funnel plots for asymmetry and conducted Egger’s regression analysis (33). Potential publication bias was further evaluated using the trim-and-fill method proposed by Duval and Tweedie (34), which estimates the number of potentially missing studies that may cause funnel plot asymmetry and imputes these studies to generate an adjusted pooled effect estimate. This method allows assessment of the robustness of the overall results after accounting for possible small-study effects or publication bias (34). A two-tailed *p* value < 0.05 was considered statistically significant. All statistical analyses were performed using RevMan (version 5.3; Cochrane Collaboration, Oxford, UK) and Stata (version 17.0; StataCorp, College Station, TX, USA).

## Results

### Database search results

**Figure 1:**
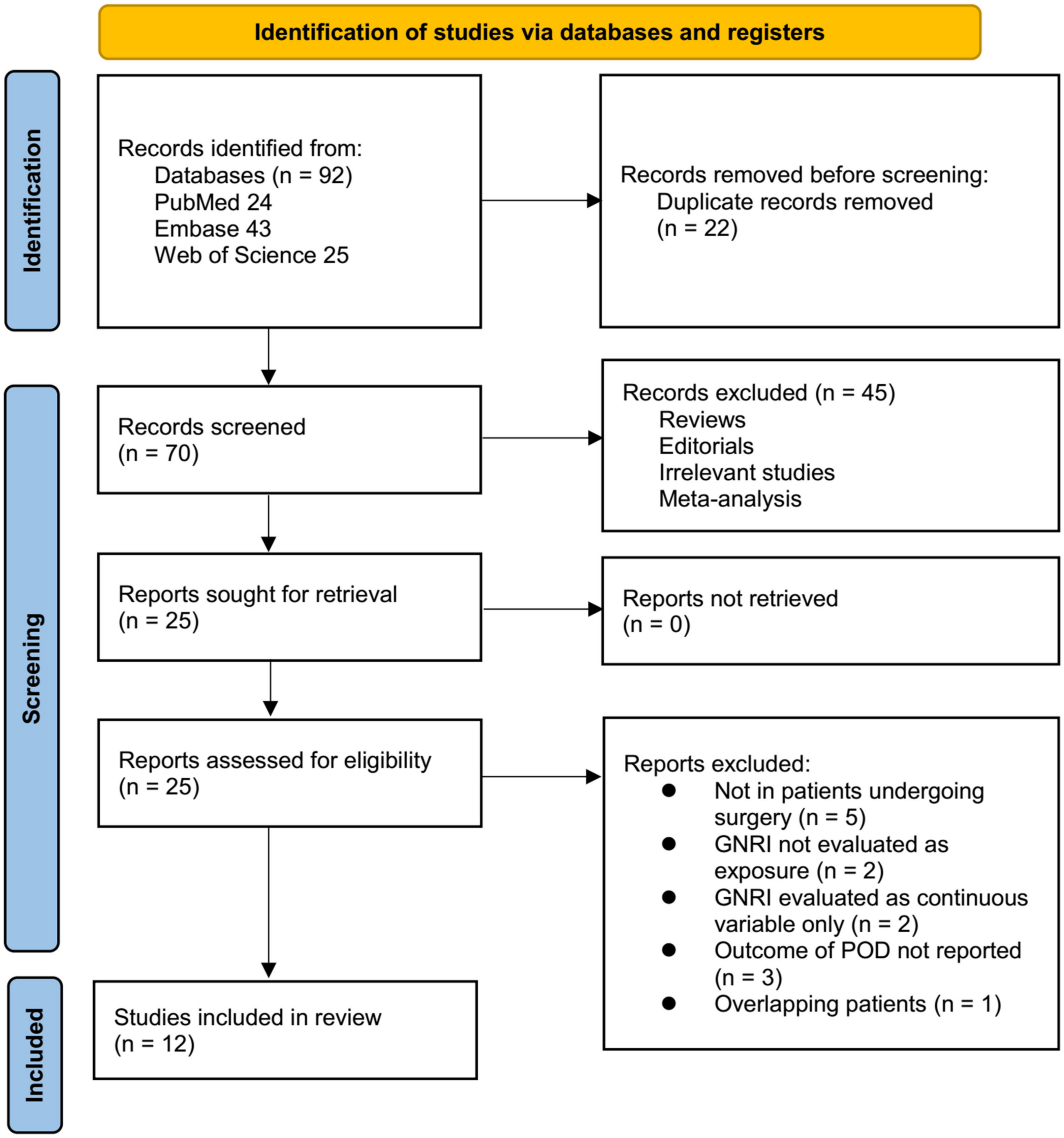
Flow diagram of the study selection process.

The study selection procedure is illustrated in **Figure 1**. A total of 92 records were retrieved from the three databases, and 22 duplicates were removed. Following screening of titles and abstracts, 45 records were excluded for failing to meet the predefined inclusion criteria. Twenty-five articles underwent full-text evaluation by two independent reviewers, after which 13 studies were excluded for the reasons detailed in **Figure 1**. Ultimately, 12 studies met the eligibility criteria and were included in the quantitative meta-analysis (15–26).

### Summary of study characteristics

The characteristics of the included studies are summarized in **Table 1**. A total of twelve cohort studies were included in this meta-analysis, comprising three prospective cohort studies (16, 22, 24) and nine retrospective cohort studies (15, 17–21, 23, 25, 26). These studies were published between 2018 and 2025 and were conducted in Asia and North America, including Japan, China, and the United States. Overall, the included studies enrolled 12,332 patients undergoing various types of surgical procedures. The mean age of participants ranged from 59.0 to 89.0 years, reflecting predominantly elderly surgical populations, and the proportion of men ranged from 37.8% to 100.0%. The included studies covered diverse surgical populations, including gastric surgery for gastric cancer, non-cardiac surgery, complete resection for non-small cell lung cancer, cardiac surgery, abdominal surgery, colorectal cancer surgery, revision hip or knee arthroplasty, laparoscopic radical prostatectomy, and multilevel thoracolumbar fusion surgery. In all studies, GNRI was assessed preoperatively, typically within one to two days before surgery or during the perioperative admission period. The cutoff values used to define low GNRI varied across studies, ranging from 82 to 101. The mean duration of postoperative follow-up ranged from 7 to 39 days. POD was diagnosed using several approaches, including validated assessment tools such as the CAM (16, 25) or the Confusion Assessment Method for the Intensive Care Unit (CAM-ICU) (19, 26) or clinical symptoms (24), or documentation based on medical records (15, 17, 18, 20–23). According, 2,090 patients (16.9%) developed POD during follow-up. Four studies (16, 18, 19, 25) adjusted for potential confounding variables such as age, sex, comorbidities, perioperative laboratory findings, and surgical characteristics, whereas the other eight studies reported unadjusted data (15, 17, 20–24, 26).

**Table 1:** Characteristics of the included studies.

Study	Country	Design	No. of patients	Mean age (years)	Men (%)	Surgery type	Timing of GNRI measurement	Cutoff of GNRI for malnutrition	Follow-up duration (days)	Diagnosis of POD	Number of patients with POD	Variables adjusted
Kushiyama 2018 (15)	Japan	RC	348	79.6	66.1	Curative gastrectomy for gastric cancer	Preoperative, 1-2 days before surgery	92	19.5	Medical records evidenced	2	None
Zhao 2020 (16)	China	PC	288	74.0	51.4	Non-cardiac surgery	Preoperative, within 2 days of admission	92	7	CAM	49	Age, sex, preoperative pain, depression, functional status by Barthel Index, and comorbidity by CCI
Takahashi 2021 (17)	Japan	RC	475	70.0	62.2	Complete resection of NSCLC	Preoperative	101	10	Medical records evidenced	17	None
Chen 2024a (18)	USA	RC	4818	59.0	45.9	Gastric surgery	Preoperative	98	10.5	Medical records evidenced	1133	Age, sex, serum calcium, sodium, blood glucose, serum potassium, SCr, BUN, renal replacement therapy, mechanical ventilation, sedatives (benzodiazepines, propofol, etc.), vasopressors, dementia, hypertension, cerebrovascular disease, diabetes, myocardial infarction, and HF
Chen 2024b (19)	USA	RC	4286	74.0	68.9	Cardiac surgery	Preoperative	98	10	CAM-ICU	659	Age, sex, ethnicity, comorbidities, alcohol abuse, preoperative lab findings (creatinine, hemoglobin, WBC), type of surgery, and postoperative factors (lab findings, vital signs, SOFA score on first ICU day, benzodiazepine use)
Nakamura 2025 (20)	Japan	RC	105	74.0	51.4	Surgery for CRC	Preoperative	94	30	Medical records evidenced	5	None
Chen 2025a (25)	China	RC	820	61.5	46.1	Revision hip or knee arthroplasty	Preoperative, within 48 hour before surgery	92	7	CAM	76	Age, sex, BMI, surgical time, length of stay, surgical site, and specific comorbidities (COPD, valvular heart disease, cerebrovascular disease, solid tumor, depression, VTE), and admission-to-surgery time
Chen 2025b (26)	China	RC	333	75.0	67.6	Abdominal surgery	Preoperative, 1-2 days before surgery	82	7	CAM-ICU	69	None
Zhou 2025 (24)	China	RC	264	71.7	100.0	Laparoscopic radical prostatectomy	Preoperative	98	7	Medical records evidenced	54	None
Teraishi 2025 (22)	Japan	PC	24	89.0	41.7	Surgery for CRC	Preoperative	98	30	Medical records evidenced	2	None
Ode 2025 (21)	Japan	RC	249	74.5	37.8	Multilevel thoracolumbar fusion surgery	Preoperative	92	39	Medical records evidenced	7	None
You 2025 (23)	China	PC	322	74.8	56.2	Radical surgery for CRC	Preoperative	98	16.8	Clinical assessment based on symptoms	17	None

RC, retrospective cohort; PC, prospective cohort; GNRI, Geriatric Nutritional Risk Index; POD, postoperative delirium; CAM, Confusion Assessment Method; CAM-ICU, Confusion Assessment Method for the Intensive Care Unit; NSCLC, non-small cell lung cancer; CRC, colorectal cancer; BMI, body mass index; COPD, chronic obstructive pulmonary disease; VTE, venous thromboembolism; CCI, Charlson Comorbidity Index; HF, heart failure; SCr, serum creatinine; BUN, blood urea nitrogen; WBC, white blood cell; SOFA, Sequential Organ Failure Assessment; ICU, intensive care unit.

### Study quality evaluation

The methodological quality of the included studies was assessed using the NOS, and the detailed results are presented in **Table 2**. The NOS scores ranged from 6 to 9, indicating generally moderate to high methodological quality among the included studies. Two studies (16, 25) achieved the highest score of 9, reflecting strong cohort representativeness, appropriate control for confounding factors, reliable outcome assessment, and adequate follow-up. One study was scored 8 due to the lack of representativeness of the exposed cohort (19). Two studies received scores of 7 due to lack of adjustment for confounding variables (23, 26) and another study also received a score of 7 due to lack of representativeness of the exposed cohort and limitations in outcome ascertainment (18). The remaining six studies scored 6 (15, 17, 20–22, 24), mainly because of limited control for important confounding factors and reliance on medical record documentation for postoperative delirium assessment. Overall, all included studies were considered to be of moderate to high methodological quality, supporting the reliability of the pooled estimates examining the association between GNRI and the risk of POD.

**Table 2:** Study quality evaluation via the Newcastle-Ottawa Scale.

Study	Representativeness of the exposed cohort	Selection of the non-exposed cohort	Ascertainment of exposure	Outcome not present at baseline	Control for age	Control for other confounding factors	Assessment of outcome	Enough long follow-up duration	Adequacy of follow-up of cohorts	Total
Kushiyama 2018 (15)	1	1	1	1	0	0	0	1	1	6
Zhao 2020 (16)	1	1	1	1	1	1	1	1	1	9
Takahashi 2021 (17)	1	1	1	1	0	0	0	1	1	6
Chen 2024a (18)	0	1	1	1	1	1	0	1	1	7
Chen 2024b (19)	0	1	1	1	1	1	1	1	1	8
Nakamura 2025 (20)	1	1	1	1	0	0	0	1	1	6
Chen 2025a (25)	1	1	1	1	1	1	1	1	1	9
Chen 2025b (26)	1	1	1	1	1	0	0	1	1	7
Zhou 2025 (24)	1	1	1	1	0	0	0	1	1	6
Teraishi 2025 (22)	1	1	1	1	0	0	0	1	1	6
Ode 2025 (21)	1	1	1	1	0	0	0	1	1	6
You 2025 (23)	1	1	1	1	0	0	1	1	1	7

### Meta-analysis results

**Figure 2:**
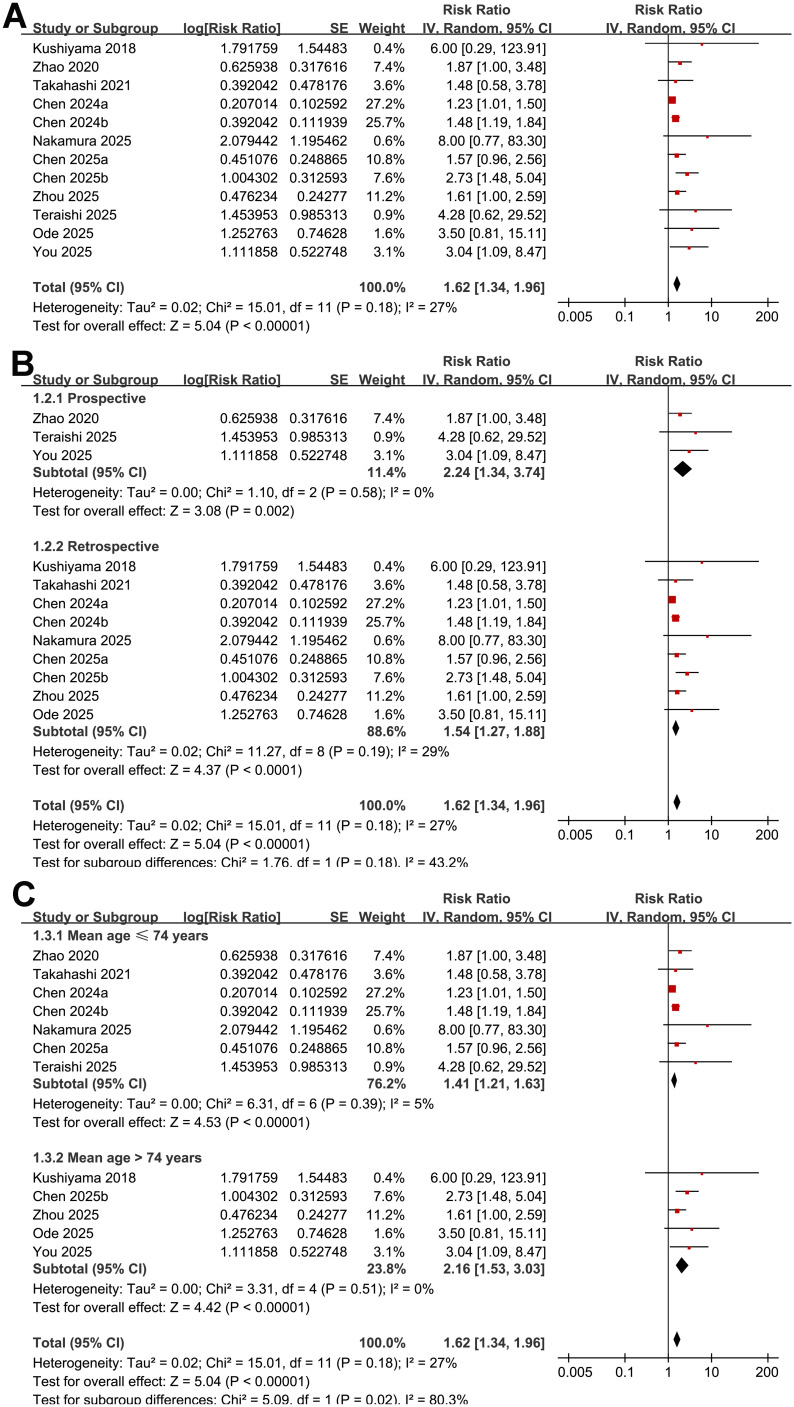
Forest plots showing the meta-analysis of the association between low GNRI and risk of POD: **(A)** overall meta-analysis; **(B)**, subgroup analysis by study design; and **(C)** subgroup analysis by mean ages of the patients.

Across 12 cohort studies (15–26), the pooled results of the meta-analysis demonstrated that malnutrition as indicated by low GNRI was associated with a significantly increased risk of POD in adult patients (RR: 1.62, 95% CI: 1.34–1.96; *p* < 0.001; **Figure 2A**) with moderate heterogeneity (*p* for Cochrane Q test = 0.18; I^2^ = 27%). Removing studies one at a time did not substantially alter the overall results, with pooled RRs ranging from 1.48 to 1.77 (all *p* < 0.05).

### Subgroup analysis results

**Figure 3:**
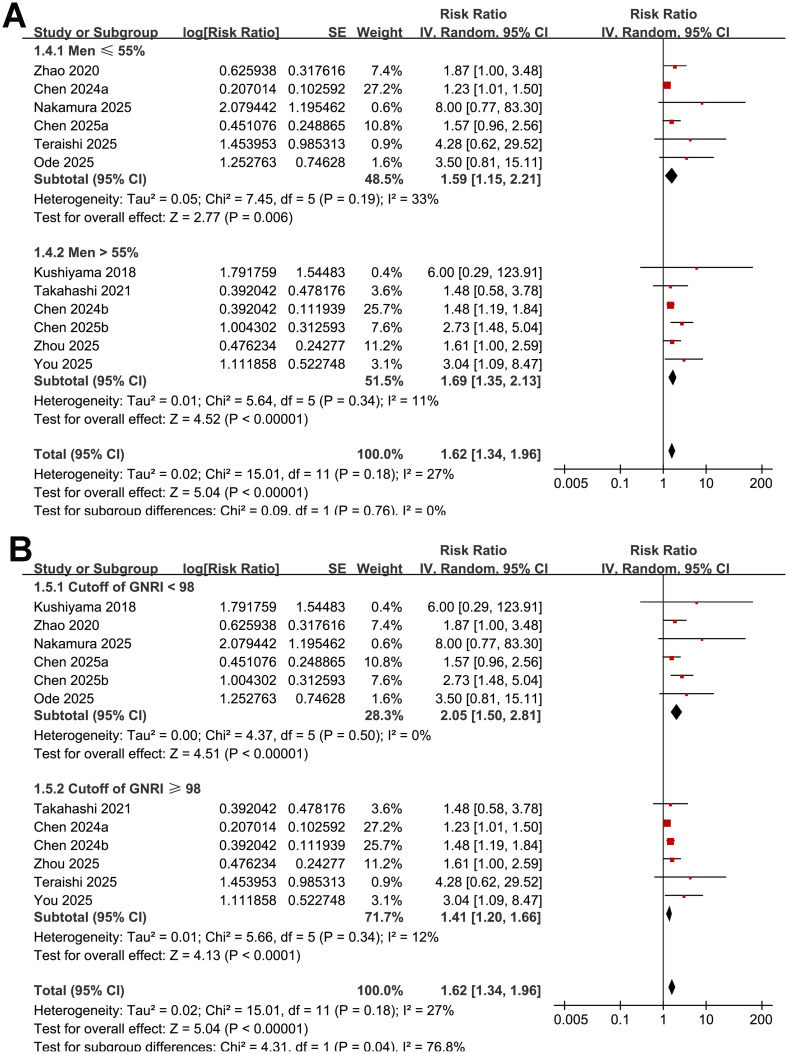
Forest plots showing the subgroup analyses of the association between low GNRI and risk of POD: **(A)** subgroup analysis by the proportions of men; and **(B)** subgroup analysis by cutoffs of GNRI for defining malnutrition.

**Figure 4:**
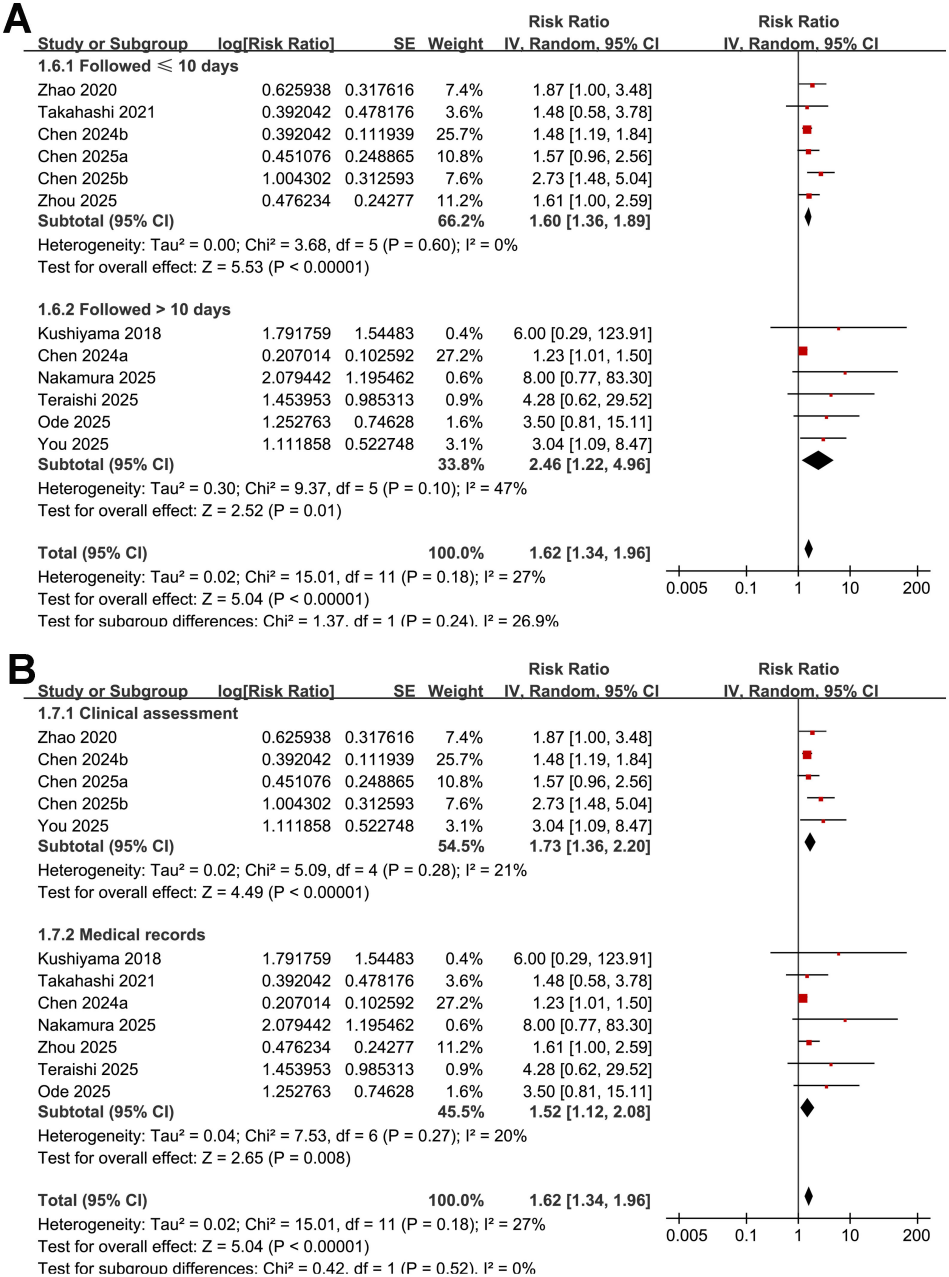
Forest plots showing the subgroup analyses of the association between low GNRI and risk of POD: **(A)** subgroup analysis by follow-up durations; and **(B)** subgroup analysis by methods for the diagnosis of POD.

**Figure 5:**
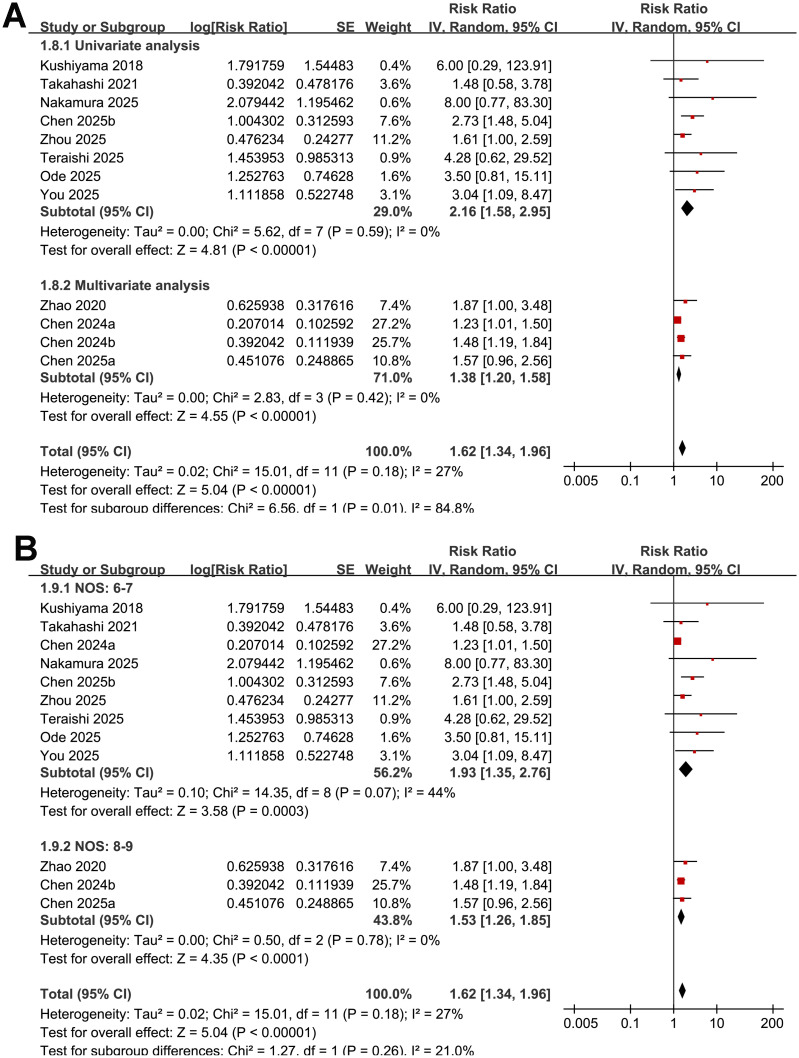
Forest plots showing the subgroup analyses of the association between low GNRI and risk of POD: **(A)** subgroup analysis by analytic models; and **(B)** subgroup analysis by study quality scores.

Further subgroup analysis suggested that the association between low GNRI and an increased risk of POD was consistent in prospective and retrospective studies (RR: 2.24 vs. 1.54; *p* for subgroup difference = 0.18; **Figure 3B**). However, a stronger association was observed in studies with the mean ages of the patients > 74 years as compared with those ≤ 74 years (RR: 2.16 vs. 1.41; *p* for subgroup difference = 0.02; **Figure 3C**). The association seemed to be consistent in studies of patients with the proportion of men ≤ 55% and > 55% (RR: 1.59 vs. 1.69; *p* for subgroup difference = 0.76; **Figure 3A**), while a stronger association was observed in studies with the cutoffs of GNRI < 98 as compared to those ≥ 98 (RR: 2.05 vs. 1.41; *p* for subgroup difference = 0.04; **Figure 3B**). The subgroup analyses showed similar results between studies with follow-up duration ≤ 10 days and > 10 days (RR: 1.60 vs. 2.46; *p* for subgroup difference = 0.24; **Figure 4A**), and in studies with POD diagnosed by clinical assessment or validated by medical records (RR: 1.73 vs. 1.52; *p* for subgroup difference = 0.52; **Figure 4B**). Although both results were statistically significant, the association was significantly weaker in studies using multivariate analysis compared with those using univariate analysis (RR: 1.38 vs. 2.16; *p* for subgroup difference = 0.01; **Figure 5A**). Heterogeneity was absent in both subgroups (I² = 0%), suggesting that differences in analytic models may contribute to the observed heterogeneity The results were not significantly different between studies with a NOS score of 6–7 and those with a NOS score of 8–9 (RR: 1.93 vs. 1.53; *p* for subgroup difference = 0.26; **Figure 5B**).

### Meta-regression analysis results

Univariate meta-regression analyses were performed to explore potential sources of heterogeneity, with the results summarized in **Table 3**. Among the examined variables, the GNRI cutoff value was significantly associated with the pooled effect estimate (*p* = 0.04) and explained most of the between-study heterogeneity (adjusted R² = 88.9%). Mean age showed a borderline association with the effect size (*p* = 0.06), accounting for 35.6% of heterogeneity. In contrast, the proportion of men, follow-up duration, and NOS score were not significantly associated with the effect estimates.

**Table 3:** Results of univariate meta-regression analysis.

Variables	RR for the association between GNRI and POD			
	Coefficient	95% CI	*p* values	Adjusted R^2^
Mean age (years)	0.027	-0.002 to 0.057	0.06	35.6%
Men (%)	0.00052	-0.01383 to 0.01487	0.94	0%
Cutoff of GNRI	-0.041	-0.081 to -0.001	0.04	88.9%
Follow-up duration (days)	0.031	-0.013 to 0.075	0.14	0%
NOS	-0.061	-0.306 to 0.184	0.59	0%

GNRI, Geriatric Nutritional Risk Index; POD, postoperative delirium; RR, risk ratio; CI, confidence interval; NOS, Newcastle-Ottawa Scale.

### Publication bias

**Figure 6:**
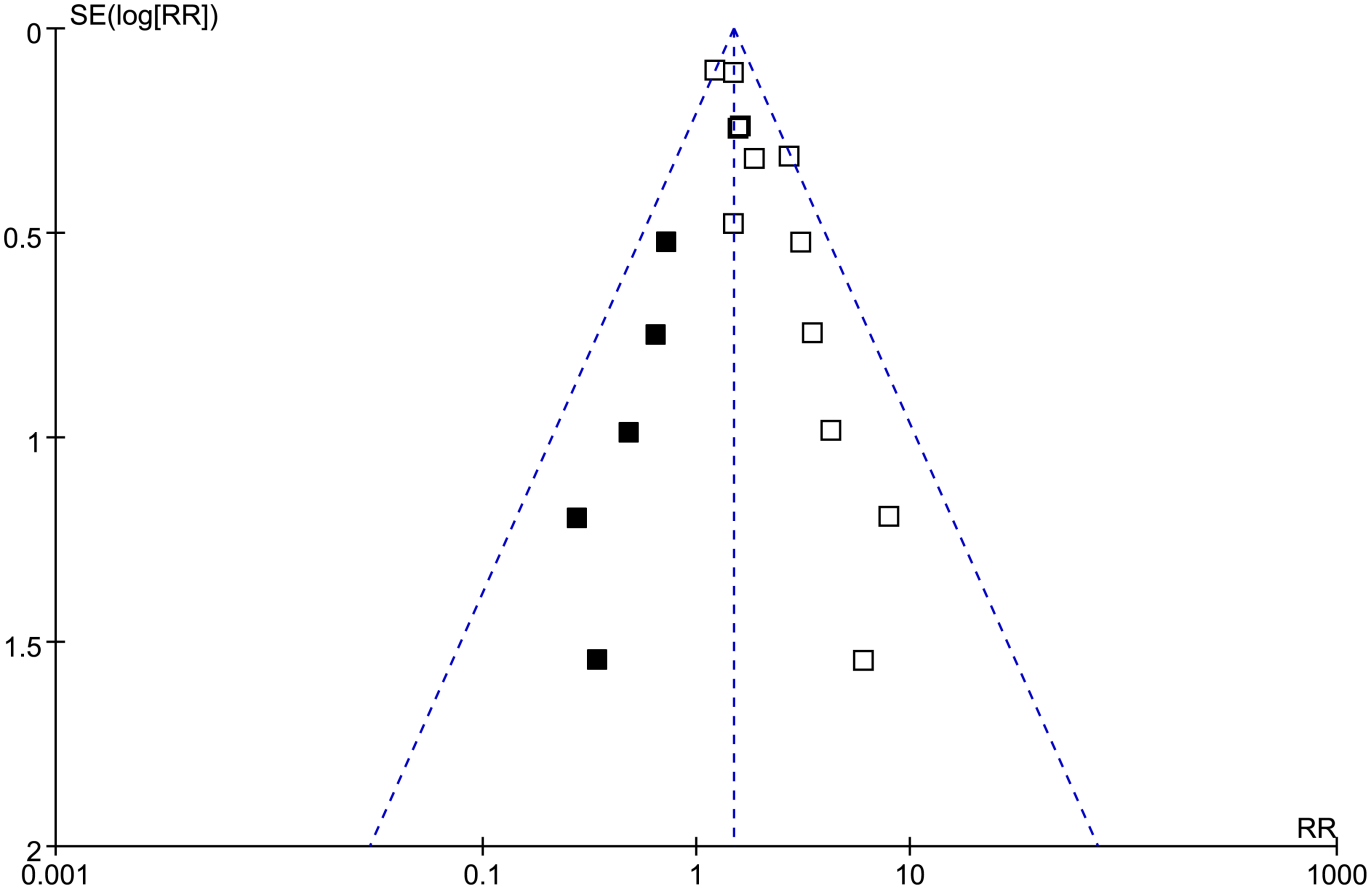
Funnel plots with “trim-and-fill” analysis for the meta-analysis of the association between low GNRI and risk of POD. White squares indicate the original studies, and black squares indicate the hypothetical studies imputed by the trim-and-fill method. The original funnel plot shows mild asymmetry, whereas the distribution becomes more symmetrical after the imputed studies are added.

As shown in **Figure 6**, visual inspection of the funnel plot suggested mild asymmetry, indicating a potential risk of publication bias. However, Egger’s regression test was not statistically significant (*p* = 0.11). Using the trim-and-fill method, five hypothetical studies were imputed, and the funnel plot became more symmetrical after their inclusion. The pooled estimate remained significant and only slightly changed from RR = 1.62 (95% CI: 1.34–1.96) before trim-and-fill to RR = 1.54 (95% CI: 1.26–1.87) after adjustment, suggesting that potential publication bias had limited influence on the overall results.

## Discussion

This meta-analysis synthesized the available cohort evidence to evaluate the association between the GNRI and POD in surgical patients. The pooled findings indicate that patients with lower GNRI, reflecting poorer nutritional status, have a higher likelihood of developing POD after surgery. The association remained generally consistent across multiple additional analyses, including subgroup, sensitivity, and trim-and-fill analyses, suggesting that the overall findings are relatively robust. Importantly, heterogeneity across studies was low to moderate, and publication bias appeared limited, as suggested by the approximately symmetrical funnel plot and the non-significant Egger’s test. These findings collectively support the concept that impaired preoperative nutritional status may be an important contributor to delirium vulnerability in surgical populations.

Compared with the previously published meta-analysis by Xie and Wu (35), which included only six studies with 4,242 patients, the present study provides a more comprehensive and methodologically rigorous synthesis of the evidence. The earlier study (35) relied mainly on crude data and in some cases included nutritional screening tools other than GNRI, such as the nutritional risk index (NRI) or Nutritional Risk Screening 2002 (NRS-2000), potentially affecting the comparability and reliability of the pooled results. In contrast, the current meta-analysis incorporated a substantially larger number of studies and participants and restricted inclusion to studies that specifically evaluated GNRI, thereby improving the clinical consistency of the exposure definition. In addition, all included studies adopted cohort designs, which allowed clearer temporal relationships between preoperative nutritional status and postoperative outcomes. Furthermore, the availability of multivariable-adjusted estimates in several studies enabled additional analyses to evaluate the robustness of the association, including subgroup analyses, meta-regression, and sensitivity analyses. These methodological improvements enhance the reliability and interpretability of the present findings.

Several biological and clinical pathways may help to interpret the observed association between GNRI and POD. However, it should be emphasized that these are hypothesis-generating considerations rather than mechanisms directly established by experimental or neuroscience-based studies. GNRI is calculated using serum albumin concentration and the ratio of actual to ideal body weight, both of which are widely recognized indicators of nutritional and physiological reserve (13, 36). Hypoalbuminemia, one of the key components of GNRI, may reflect systemic inflammation, impaired protein synthesis, and reduced physiological reserve (37). Albumin also plays important roles in maintaining oncotic pressure, transporting endogenous and exogenous substances, and exerting antioxidant and anti-inflammatory effects (37, 38). Reduced albumin levels may therefore contribute to systemic inflammatory activation and increased blood–brain barrier permeability, which have been implicated in the pathogenesis of delirium (39, 40). In addition, low body weight relative to ideal weight may reflect chronic energy deficiency, sarcopenia, and frailty, all of which have been associated with increased vulnerability to perioperative stress (41, 42). Malnutrition may also impair neurotransmitter synthesis, mitochondrial function, and immune responses, thereby promoting neuroinflammation and neuronal dysfunction during the perioperative period (12). In addition, inflammation, oxidative stress, and metabolic dysregulation—processes commonly linked to malnutrition—have been implicated in the pathophysiology of delirium in broader literature (43, 44). However, direct evidence specifically connecting GNRI-defined malnutrition to these neurobiological pathways in POD remains limited. Therefore, the above mechanisms should be interpreted cautiously as plausible explanations rather than confirmed causal pathways,. The GNRI is a composite clinical index rather than a biological mediator, and its association with POD likely reflects the combined effects of underlying nutritional and physiological deficits rather than a direct mechanistic role. Therefore, the pathways discussed above are inferred from general evidence on malnutrition and delirium and should not be interpreted as GNRI-specific mechanisms. Future experimental and translational studies are needed to clarify the biological links between nutritional status, as captured by GNRI, and the neurobiology of delirium.

The subgroup and meta-regression analyses of this meta-analysis provide further insights into potential sources of heterogeneity among studies. First, the association between GNRI and POD appeared stronger in studies with older populations, suggesting that age may modify the relationship between nutritional status and delirium risk. Advanced age is a well-established risk factor for POD and is frequently incorporated into predictive models for postoperative delirium (45, 46). Older patients are more likely to experience malnutrition, frailty, sarcopenia, and multimorbidity, which may amplify the deleterious effects of poor nutritional status on brain vulnerability (47). Second, differences in GNRI cutoff values across studies significantly influenced the pooled effect size. Lower cutoff thresholds may identify patients with more severe nutritional impairment, which could be associated with a higher risk of postoperative complications, including delirium. Therefore, variability in the definition of “low GNRI” across studies may contribute to between-study heterogeneity. Third, the analytic model used in the original studies also affected the observed association. Studies reporting multivariate-adjusted estimates generally showed weaker associations compared with those based on univariate analyses, suggesting that part of the crude association between GNRI and POD may be explained by confounding factors such as age, comorbidities, and surgical characteristics. Although the association remained statistically significant in analyses accounting for selected confounders, the attenuation of effect sizes in multivariate models suggests that part of the observed relationship may be explained by underlying factors such as age, frailty, comorbidities, or systemic inflammation. Therefore, GNRI may primarily function as a marker of overall vulnerability rather than an independent causal determinant of POD.

Several limitations of this meta-analysis should also be acknowledged. First, although all included studies adopted cohort designs, some were retrospective in nature, which may introduce selection bias and recall bias (48). Second, the included studies were subject to several sources of clinical and methodological heterogeneity, including differences in study design (predominantly retrospective cohorts), surgical populations, and methods used for POD ascertainment (e.g., validated tools such as CAM versus diagnoses based on medical records). These variations may introduce selection and detection biases that cannot be fully addressed through statistical modeling. Although we applied random-effects models and conducted subgroup and meta-regression analyses to explore potential sources of heterogeneity, these approaches can only partially mitigate the influence of such biases. Notably, our analyses suggested that mean age and GNRI cutoff values contributed to between-study variability, whereas differences in study design and POD assessment methods did not significantly modify the pooled association. Nevertheless, these findings should be interpreted with caution given the observational nature of the included studies. Besides, it should be noted that the subgroup and meta-regression analyses were exploratory in nature. The use of median-based cutoffs may not reflect clinically meaningful thresholds, and the performance of multiple comparisons increases the risk of false-positive findings. Therefore, these results should be interpreted cautiously and considered hypothesis-generating rather than confirmatory. Third, residual confounding cannot be fully excluded, as important factors such as baseline cognitive status, frailty severity, inflammatory burden, and perioperative management were not consistently adjusted for across studies. Alternative explanations should also be considered. For example, low GNRI may reflect shared underlying frailty or disease severity that predisposes patients to delirium, rather than exerting a direct effect. Reverse causation is also possible, whereby patients with preclinical vulnerability or early cognitive impairment may have poorer nutritional status prior to surgery. These considerations further support cautious interpretation of the observed associations. Moreover, factors such as preexisting cognitive impairment, medication use, or perioperative complications may also influence the risk of delirium but were not uniformly reported across studies (49). Fourth, the pooled estimate may have been influenced by a small number of relatively large studies, including database-based cohorts, which contributed greater statistical weight to the analysis. Although leave-one-out sensitivity analyses did not identify any single study as having a decisive impact on the overall results, this approach may not fully account for the cumulative influence of large studies. To further assess robustness, trim-and-fill analysis was performed, which imputed several potentially missing studies with smaller or null effects. The adjusted pooled estimate remained largely unchanged, suggesting that the observed association was not solely driven by the largest studies. Nevertheless, the potential influence of study size and weighting should be considered when interpreting the results. Another important limitation relates to the pooling of effect estimates derived from both univariate and multivariate analyses. Studies reporting only unadjusted estimates were included to avoid excluding potentially relevant data, particularly as some of these studies reported non-significant associations. However, unadjusted estimates are more susceptible to confounding and may overestimate the strength of the association. Consistent with this concern, our subgroup analysis showed attenuated effect sizes in studies using multivariate models compared with those using univariate analyses. Therefore, the overall pooled estimate likely reflects a balance between adjusted and unadjusted data, and the magnitude of the association should be interpreted with caution. Future studies providing fully adjusted estimates would help to clarify the independent relationship between GNRI and POD. Furthermore, the potential modifying effect of surgical type or procedural complexity could not be adequately evaluated. The included studies encompassed a wide range of surgical procedures with substantial heterogeneity, and detailed information on operative characteristics such as surgical duration, blood loss, inflammatory response, or postoperative ICU exposure was not consistently reported. As a result, it was not possible to stratify analyses according to surgical stress or complexity at the study level. Future studies with more granular perioperative data are needed to clarify whether the association between GNRI and POD differs according to surgical burden. Finally, as with all meta-analyses of observational studies, the findings should be interpreted as evidence of association rather than causation.

Despite these limitations, the findings of this study may have potential implications for risk stratification rather than direct clinical decision-making. GNRI is a simple, inexpensive, and routinely available index derived from laboratory and anthropometric measurements, making it feasible for use in preoperative assessment (36). However, the present study did not compare GNRI with other nutritional assessment measures, and therefore no conclusions can be drawn regarding its relative performance or superiority. Future studies directly comparing GNRI with other established nutritional indices (such as the Prognostic Nutritional Index or the Controlling Nutritional Status) are needed to better define Moreover, given the observational nature of the included studies, the present results should be interpreted as evidence of association rather than causation. While the findings do not establish that GNRI-guided interventions can prevent POD, they are consistent with the broader recognition that poor nutritional status is associated with adverse surgical outcomes and should be routinely assessed and managed in clinical practice. In this context, GNRI may serve as a pragmatic marker to help identify patients at higher risk who may benefit from closer perioperative monitoring and comprehensive assessment. Future prospective and interventional studies are required to determine whether incorporating GNRI into risk stratification or nutritional optimization strategies can reduce the incidence of POD.

## Conclusions

In conclusion, the present meta-analysis indicates that lower GNRI is associated with a higher risk of POD in surgical patients. While the association appears consistent across studies, the observational nature of the evidence and potential residual confounding warrant cautious interpretation. These findings support the importance of nutritional assessment in perioperative care, but the specific role of GNRI in guiding targeted interventions for POD prevention remains uncertain. Further well-designed prospective and interventional studies are needed to clarify whether improving nutritional status, particularly when guided by GNRI, can influence the risk of POD.

## Data Availability

The original contributions presented in the study are included in the article/**Supplementary Material**. Further inquiries can be directed to the corresponding author.

## References

[1] Ding XY Zhang MH Liu J Wu D . Recent advances in postoperative delirium in elderly patients: pathophysiological mechanisms, risk prediction, and therapeutic strategies. Front Neurosci. (2026) 20:1759910. doi: 10.3389/fnins.2026.1759910. PMID: 41725849 PMC12920445

[2] Thedim M Vacas S . Postoperative delirium and the older adult: Untangling the confusion. J Neurosurg Anesthesiol. (2024) 36:184–9. doi: 10.1097/ana.0000000000000971. PMID: 38683185 PMC11345733

[3] Oh ES Fong TG Hshieh TT Inouye SK . Delirium in older persons: Advances in diagnosis and treatment. JAMA. (2017) 318:1161–74. doi: 10.1002/9781118799574.ch14. PMID: 28973626 PMC5717753

[4] Fenta E Teshome D Kibret S Hunie M Tiruneh A Belete A . Incidence and risk factors of postoperative delirium in elderly surgical patients 2023. Sci Rep. (2025) 15:1400. doi: 10.1038/s41598-024-84554-2. PMID: 39789093 PMC11718272

[5] Swarbrick CJ Partridge JSL . Evidence-based strategies to reduce the incidence of postoperative delirium: A narrative review. Anaesthesia. (2022) 77:92–101. doi: 10.1111/anae.15607. PMID: 35001376

[6] Han S Zhang H Li F Hou D Lv X Lou J . Associations of postoperative delirium with perioperative frailty worsening and their combined effect on 1-year mortality in older surgical patients: A prospective cohort study. Eur Geriatr Med. (2026). doi: 10.1007/s41999-026-01423-z. PMID: 41784869 PMC13309383

[7] Goldberg TE Chen C Wang Y Jung E Swanson A Ing C . Association of delirium with long-term cognitive decline: A meta-analysis. JAMA Neurol. (2020) 77:1373–81. doi: 10.1001/jamaneurol.2020.2273. PMID: 32658246 PMC7358977

[8] Yan E Veitch M Saripella A Alhamdah Y Butris N Tang-Wai DF . Association between postoperative delirium and adverse outcomes in older surgical patients: A systematic review and meta-analysis. J Clin Anesth. (2023) 90:111221. doi: 10.1016/j.jclinane.2023.111221. PMID: 37515876

[9] Bramley P McArthur K Blayney A McCullagh I . Risk factors for postoperative delirium: An umbrella review of systematic reviews. Int J Surg. (2021) 93:106063. doi: 10.1016/j.ijsu.2021.106063. PMID: 34411752

[10] Dong B Wang J Li P Li J Liu M Zhang H . The impact of preoperative malnutrition on postoperative delirium: A systematic review and meta-analysis. Perioper Med (Lond). (2023) 12:55. doi: 10.1186/s13741-023-00345-9. PMID: 37884977 PMC10604920

[11] Dammavalam V Murphy J Johnkutty M Elias M Corn R Bergese S . Perioperative cognition in association with malnutrition and frailty: A narrative review. Front Neurosci. (2023) 17:1275201. doi: 10.3389/fnins.2023.1275201. PMID: 38027517 PMC10651720

[12] Luo L Gong Q He M Zhu Y Yu W Gong T . Bridging nutrition and neurology: Malnutrition's role in perioperative neurocognitive disorders. Front Nutr. (2025) 12:1601021. doi: 10.3389/fnut.2025.1601021. PMID: 40880745 PMC12380589

[13] Abd-El-Gawad WM Abou-Hashem RM El Maraghy MO Amin GE . The validity of geriatric nutrition risk index: Simple tool for prediction of nutritional-related complication of hospitalized elderly patients. Comparison with mini nutritional assessment. Clin Nutr. (2014) 33:1108–16. doi: 10.1016/j.clnu.2013.12.005. PMID: 24418116

[14] Luo P Shi K Luo Y Ren HB . Prognostic value of the geriatric nutritional risk index in patients undergoing cardiac surgery: A systematic review and meta-analysis. Front Nutr. (2025) 12:1628671. doi: 10.3389/fnut.2025.1628671. PMID: 40786692 PMC12331503

[15] Kushiyama S Sakurai K Kubo N Tamamori Y Nishii T Tachimori A . The preoperative geriatric nutritional risk index predicts postoperative complications in elderly patients with gastric cancer undergoing gastrectomy. In Vivo. (2018) 32:1667–72. doi: 10.21873/invivo.11430. PMID: 30348732 PMC6365747

[16] Zhao Y Ge N Xie D Gao L Wang Y Liao Y . The geriatric nutrition risk index versus the mini-nutritional assessment short form in predicting postoperative delirium and hospital length of stay among older non-cardiac surgical patients: A prospective cohort study. BMC Geriatr. (2020) 20:107. doi: 10.1186/s12877-020-1501-8. PMID: 32183760 PMC7077017

[17] Takahashi M Sowa T Tokumasu H Gomyoda T Okada H Ota S . Comparison of three nutritional scoring systems for outcomes after complete resection of non-small cell lung cancer. J Thorac Cardiovasc Surg. (2021) 162:1257–68:e3. doi: 10.1016/j.jtcvs.2020.06.030. PMID: 32771232

[18] Chen Y Chen H Zhuang Y Wang Y Dai Z . Association between the geriatric nutritional risk index and postoperative delirium in gastric surgery patients: An analysis of the MIMIC-IV database. BMC Anesthesiol. (2024) 24:477. doi: 10.1186/s12871-024-02874-2. PMID: 39731004 PMC11673328

[19] Chen Z Hao Q Sun R Zhang Y Fu H Liu S . Predictive value of the geriatric nutrition risk index for postoperative delirium in elderly patients undergoing cardiac surgery. CNS Neurosci Ther. (2024) 30:e14343. doi: 10.1111/cns.14343. PMID: 37408469 PMC10848042

[20] Nakamura Y Nishimura T Kanemitsu E Nagata H Komori J Takada Y . Usefulness of the geriatric nutritional risk index (GNRI) as a predictor of postoperative complications after colorectal cancer surgery. Cureus. (2025) 17:e86268. doi: 10.7759/cureus.86268. PMID: 40688986 PMC12275500

[21] Ode Y Segi N Ito S Ouchida J Yamauchi I Nagatani Y . Impact of nutritional risk on complications and recovery in the older people: A geriatric nutritional risk index-based study. Spine Surg Relat Res. (2025) 9:552–8. doi: 10.22603/ssrr.2025-0016. PMID: 41098610 PMC12519118

[22] Teraishi F Utsumi M Yoshida Y Shoji R Kanaya N Matsumi Y . The geriatric nutritional risk index: A key indicator of perioperative outcome in oldest-old patients with colorectal cancer. In Vivo. (2025) 39:2810–7. doi: 10.21873/invivo.14080. PMID: 40877149 PMC12396066

[23] You X Xu Y Zhao J Crippa J Lim AC Nemeth ZH . The association of geriatric nutritional risk and perioperative anesthesia-related adverse reactions in elderly patients with colorectal cancer-a prospective study. Transl Cancer Res. (2025) 14:7419–27. doi: 10.21037/tcr-2025-1140. PMID: 41234882 PMC12605544

[24] Zhou R Zhou Y Yue X Wang M Zhang Y Liu C . The prognostic implications of the geriatric nutritional risk index in patients with prostate cancer: A single-center retrospective cohort study. Healthcare (Basel). (2025) 13. doi: 10.3390/healthcare13243266. PMID: 41464335 PMC12732814

[25] Chen X Yao W Liu X Xie Q Wang D Xu H . Preoperative geriatric nutritional risk index as a predictor of postoperative delirium in revision arthroplasty: A 10-year retrospective cohort study. Front Med (Lausanne). (2025) 12:1626383. doi: 10.3389/fmed.2025.1626383. PMID: 40740942 PMC12307334

[26] Chen C Li Y Zhou D Yang Y Zhang L Wang X . Comparative predictive value of preoperative GNRI, PNI, and CONUT for postoperative delirium in geriatric abdominal surgery patients admitted to the ICU. Front Nutr. (2025) 12:1669159. doi: 10.3389/fnut.2025.1669159. PMID: 41132554 PMC12540140

[27] Page MJ Moher D Bossuyt PM Boutron I Hoffmann TC Mulrow CD . PRISMA 2020 explanation and elaboration: Updated guidance and exemplars for reporting systematic reviews. BMJ. (2021) 372:n160. doi: 10.31222/osf.io/gwdhk. PMID: 33781993 PMC8005925

[28] Higgins J Thomas J Chandler J Cumpston M Li T Page M . Cochrane handbook for systematic reviews of interventions version 6.2. In: The Cochrane Collaboration London UK (2021). Available online at: https://www.training.cochrane.org/handbook.

[29] Wells GA Shea B O'Connell D Peterson J Welch V Losos M . The Newcastle-Ottawa Scale (NOS) for assessing the quality of nonrandomised studies in meta-analyses (2010). Available online at: http://www.ohri.ca/programs/clinical_epidemiology/oxford.asp (Accessed January 22, 2026).

[30] Zhang J Yu KF . What's the relative risk? A method of correcting the odds ratio in cohort studies of common outcomes. JAMA. (1998) 280:1690–1. doi: 10.1001/jama.282.6.529-a. PMID: 9832001

[31] Higgins JP Thompson SG . Quantifying heterogeneity in a meta-analysis. Stat Med. (2002) 21:1539–58. doi: 10.1191/026921699666884611. PMID: 12111919

[32] Marušić MF Fidahić M Cepeha CM Farcaş LG Tseke A Puljak L . Methodological tools and sensitivity analysis for assessing quality or risk of bias used in systematic reviews published in the high-impact anesthesiology journals. BMC Med Res Methodol. (2020) 20:121. 32423382 10.1186/s12874-020-00966-4 PMC7236513

[33] Egger M Davey Smith G Schneider M Minder C . Bias in meta-analysis detected by a simple, graphical test. BMJ. (1997) 315:629–34. doi: 10.1136/bmj.315.7109.629. PMID: 9310563 PMC2127453

[34] Duval S Tweedie R . Trim and fill: A simple funnel-plot-based method of testing and adjusting for publication bias in meta-analysis. Biometrics. (2000) 56:455–63. doi: 10.1007/978-1-4020-8486-7_10. PMID: 10877304

[35] Xie S Wu Q . Geriatric nutritional risk index predicts postoperative delirium in elderly: A meta-analysis. Saudi Med J. (2024) 45:869–75. doi: 10.15537/smj.2024.45.9.20240216. PMID: 39218460 PMC11376695

[36] Cereda E Pedrolli C . The geriatric nutritional risk index. Curr Opin Clin Nutr Metab Care. (2009) 12:1–7. doi: 10.1097/mco.0b013e3283186f59. PMID: 19057180

[37] Soeters PB Wolfe RR Shenkin A . Hypoalbuminemia: Pathogenesis and clinical significance. JPEN J Parenter Enteral Nutr. (2019) 43:181–93. doi: 10.1002/jpen.1451. PMID: 30288759 PMC7379941

[38] Wu N Liu T Tian M Liu C Ma S Cao H . Albumin, an interesting and functionally diverse protein, varies from 'native' to 'effective' (Review). Mol Med Rep. (2024) 29. doi: 10.3892/mmr.2023.13147. PMID: 38099350 PMC10784728

[39] Hillmer L Erhardt EB Caprihan A Adair JC Knoefel JE Prestopnik J . Blood-brain barrier disruption measured by albumin index correlates with inflammatory fluid biomarkers. J Cereb Blood Flow Metab. (2023) 43:712–9. doi: 10.1016/j.cccb.2024.100312. PMID: 36522849 PMC10108191

[40] Simone MJ Tan ZS . The role of inflammation in the pathogenesis of delirium and dementia in older adults: A review. CNS Neurosci Ther. (2011) 17:506–13. doi: 10.1111/j.1755-5949.2010.00173.x. PMID: 20553303 PMC6493838

[41] Curtis M Swan L Fox R Warters A O'Sullivan M . Associations between body mass index and probable sarcopenia in community-dwelling older adults. Nutrients. (2023) 15. doi: 10.3390/nu15061505. PMID: 36986233 PMC10059806

[42] Gao Y Zhang H Fang K Yao Y Chen J Lu H . The relationship between frailty, BMI, and mortality in older adults: Results from the CLHLS. BMC Geriatr. (2025) 25:539. doi: 10.1186/s12877-025-06197-w. PMID: 40684122 PMC12275446

[43] Piccirillo A Perri F Vittori A Ionna F Sabbatino F Ottaiano A . Evaluating nutritional risk factors for delirium in intensive-care-unit patients: Present insights and prospects for future research. Clin Pract. (2023) 13:1577–92. doi: 10.3390/clinpract13060138. PMID: 38131687 PMC10742123

[44] El Sheikh WG Sleem B Kobeissy F Bizri M . Biomarkers of delirium and relation to dementia among the elderly in the intensive care unit: A narrative review. biomark Neuropsychiatr. (2023) 8:100064. doi: 10.1016/j.bionps.2023.100064. PMID: 41916819

[45] Xu MM Zhou JJ Han MT Sun T Hui KL . Analysis of risk factors and development of a predictive model for postoperative delirium in elderly patients undergoing cardiac surgery with general anesthesia. Med (Baltimore). (2025) 104:e46021. doi: 10.1097/md.0000000000046021. PMID: 41305787 PMC12643621

[46] Song Y Zhang D Wang Q Liu Y Chen K Sun J . Prediction models for postoperative delirium in elderly patients with machine-learning algorithms and SHapley additive explanations. Transl Psychiatry. (2024) 14:57. doi: 10.1038/s41398-024-02762-w. PMID: 38267405 PMC10808214

[47] İncedal Irgat S Kızıltan G . Associations between frailty, sarcopenia, and nutritional status in older adults living in nursing homes. Nutrients. (2025) 17. doi: 10.3390/nu17223574. PMID: 41305625 PMC12655629

[48] Pannucci CJ Wilkins EG . Identifying and avoiding bias in research. Plast Reconstr Surg. (2010) 126:619–28. doi: 10.1097/prs.0b013e3181de24bc. PMID: 20679844 PMC2917255

[49] Paunikar S Chakole V . Postoperative delirium and neurocognitive disorders: A comprehensive review of pathophysiology, risk factors, and management strategies. Cureus. (2024) 16:e68492. doi: 10.7759/cureus.68492. PMID: 39364454 PMC11447296

