# Supplementary material for: Association between the geriatric nutritional risk index and postoperative delirium: a meta-analysis
Source: Front Psychiatry. 2026 Apr 23;17:1826824. doi: 10.3389/fpsyt.2026.1826824 (PMC13149375; doi:10.3389/fpsyt.2026.1826824)
Supplement: Supplementary file 1 [file Table1.docx]

**Detailed search strategy for each database**

**PubMed**

#1 "Geriatric Nutritional Risk Index"[tiab] OR "Geriatric Nutrition Risk Index"[tiab] OR GNRI[tiab]

#2 "Surgical Procedures, Operative"[Mesh] OR surgery[tiab] OR surgical[tiab] OR postoperative[tiab] OR post-operative[tiab] OR postsurgical[tiab] OR post-surgical[tiab]

#3 "Delirium"[Mesh] OR delirium[tiab] OR confusion[tiab] OR "acute encephalopathy"[tiab] OR "cognitive dysfunction"[tiab] OR "cognitive impairment"[tiab] OR "cognitive disorder"[tiab] OR "altered mental status"[tiab] OR "organic brain syndrome"[tiab]

#4 #1 AND #2 AND #3

**Embase**

#1 'geriatric nutritional risk index':ti,ab OR 'geriatric nutrition risk index':ti,ab OR gnri:ti,ab

#2 'surgery'/exp OR surgery:ti,ab OR surgical:ti,ab OR postoperative:ti,ab OR 'post operative':ti,ab OR postsurgical:ti,ab

#3 'delirium'/exp OR delirium:ti,ab OR confusion:ti,ab OR 'acute encephalopathy':ti,ab OR 'cognitive dysfunction':ti,ab OR 'cognitive impairment':ti,ab OR 'cognitive disorder':ti,ab OR 'altered mental status':ti,ab OR 'organic brain syndrome':ti,ab

#4 #1 AND #2 AND #3

**Web of Science**

TS=("geriatric nutritional risk index" OR "geriatric nutrition risk index" OR GNRI) AND TS=(surgery OR surgical OR postoperative OR "post-operative" OR postsurgical OR "post-surgical") AND TS=(confusion OR delirium OR "acute encephalopathy" OR "cognitive dysfunction" OR "cognitive impairment" OR "cognitive disorder" OR "altered mental status" OR "organic brain syndrome")
